# Supplementary material for: Modeling glioblastoma heterogeneity as a dynamic network of cell states
Source: Mol Syst Biol. 2021 Sep 16;17(9):e10105. doi: 10.15252/msb.202010105 (PMC8444284; doi:10.15252/msb.202010105)
Supplement: Supplementary file 6 — Source Data for Figure 5 [file MSB-17-e10105-s004.zip › Figure5A_sourcedata/GSEA_3017/hallmarks_stateE.GseaPreranked.1621934677158/HALLMARK_DNA_REPAIR.html]

Details for gene set HALLMARK\_DNA\_REPAIR[GSEA]

|  || Dataset | state63017 |
| Phenotype | NoPhenotypeAvailable |
| Upregulated in class | na\_neg |
| GeneSet | HALLMARK\_DNA\_REPAIR |
| Enrichment Score (ES) | -0.38183278 |
| Normalized Enrichment Score (NES) | -1.5251153 |
| Nominal p-value | 0.05419355 |
| FDR q-value | 0.06803544 |
| FWER p-Value | 0.475 |
Table: GSEA Results Summary

  

Fig 1: Enrichment plot: HALLMARK\_DNA\_REPAIR      
 Profile of the Running ES Score & Positions of GeneSet Members on the Rank Ordered List

  

| PROBE | GENE SYMBOL | GENE\_TITLE | RANK IN GENE LIST | RANK METRIC SCORE | RUNNING ES | CORE ENRICHMENT || 1 | CETN2 |  |  | 145 | 0.338 | -0.1307 | No |
| 2 | NME3 |  |  | 185 | 0.308 | -0.1485 | No |
| 3 | GUK1 |  |  | 231 | 0.277 | -0.1754 | No |
| 4 | IMPDH2 |  |  | 351 | -0.269 | -0.2834 | No |
| 5 | POLR2A |  |  | 361 | -0.272 | -0.2715 | No |
| 6 | ADA |  |  | 371 | -0.273 | -0.2594 | No |
| 7 | REV3L |  |  | 430 | -0.292 | -0.2991 | No |
| 8 | UPF3B |  |  | 479 | -0.308 | -0.3268 | No |
| 9 | RFC2 |  |  | 498 | -0.313 | -0.3214 | No |
| 10 | COX17 |  |  | 505 | -0.316 | -0.3027 | No |
| 11 | POLA2 |  |  | 521 | -0.320 | -0.2934 | No |
| 12 | RFC5 |  |  | 534 | -0.326 | -0.2804 | No |
| 13 | PRIM1 |  |  | 539 | -0.328 | -0.2585 | No |
| 14 | RAD51 |  |  | 553 | -0.335 | -0.2458 | No |
| 15 | SAC3D1 |  |  | 679 | -0.416 | -0.3486 | Yes |
| 16 | RPA2 |  |  | 688 | -0.424 | -0.3234 | Yes |
| 17 | POLD3 |  |  | 710 | -0.437 | -0.3113 | Yes |
| 18 | ALYREF |  |  | 716 | -0.444 | -0.2813 | Yes |
| 19 | POLA1 |  |  | 733 | -0.460 | -0.2619 | Yes |
| 20 | LIG1 |  |  | 752 | -0.485 | -0.2427 | Yes |
| 21 | RFC4 |  |  | 777 | -0.527 | -0.2266 | Yes |
| 22 | RPA3 |  |  | 788 | -0.550 | -0.1936 | Yes |
| 23 | RFC3 |  |  | 801 | -0.569 | -0.1611 | Yes |
| 24 | PCNA |  |  | 832 | -0.640 | -0.1426 | Yes |
| 25 | ZWINT |  |  | 847 | -0.692 | -0.1025 | Yes |
| 26 | FEN1 |  |  | 862 | -0.736 | -0.0589 | Yes |
| 27 | DUT |  |  | 903 | -0.887 | -0.0315 | Yes |
| 28 | TYMS |  |  | 917 | -0.965 | 0.0316 | Yes |
Table: GSEA details [plain text format]

  

Fig 2: HALLMARK\_DNA\_REPAIR: Random ES distribution      
 Gene set null distribution of ES for **HALLMARK\_DNA\_REPAIR**
